# High Expression of DC-STAMP Gene Predicts Adverse Outcomes in AML

**DOI:** 10.3389/fgene.2022.876689

**Published:** 2022-04-27

**Authors:** Qian Liang, Lele Zhang, Wenjun Wang, Jingyu Zhao, Qiaoli Li, Hong Pan, Zhen Gao, Liwei Fang, Jun Shi

**Affiliations:** Regenerative Medicine Clinic, State Key Laboratory of Experimental Hematology, National Clinical Research Center for Blood Diseases, Haihe Laboratory of Cell Ecosystem, Institute of Hematology & Blood Diseases Hospital, Chinese Academy of Medical Sciences & Peking Union Medical College, Tianjin, China

**Keywords:** DC-STAMP, acute myeloid leukemia, prognosis, bioinformatics, TCGA, immune checkpoints

## Abstract

Acute myeloid leukemia (AML) is a genetically heterogeneous hematological malignancy with poor prognosis. We explored the RNA sequence data and clinical information of AML patients from The Cancer Genome Atlas (TCGA) and Genotype-Tissue Expression (GTEx) database to search for the core molecule for prognosis. The *DC-STAMP* expression was significantly higher in AML patients, which was linked to old age, unfavorable cytogenetic risk, and death (all *p* < 0.05). Furthermore, it was revealed that high *DC-STAMP* expression was an independent unfavorable factor for overall survival (OS) by univariate analysis [hazard ratio (HR): 2.683; 95% confidence interval (CI): 1.723–4.178; *p* < 0.001] and multivariate analysis (HR: 1.733; 95% CI: 1.079–2.781; *p* = 0.023). The concordance index (C-index 0.734, 95% CI: 0.706–0.762), calibration curves, and decision curve analysis showed the certain predictive accuracy of a nomogram model based on multivariate analysis for OS. In addition, we found that the differentially expressed gene (DEG) enrichment pathways of high- and low-*DC-STAMP* expression group enrichment pathways were focused on channel activity and platelet alpha granule by the Gene Ontology (GO) and Kyoto Encyclopedia of Genes and Genomes (KEGG), while gene set enrichment analysis (GSEA) pathways were mainly involved in mTORC1 signaling and TNF-α signaling *via* the NF-kB pathway. Moreover, a protein–protein interaction (PPI) network demonstrated that *DC-STAMP* interacted with two hub genes (*PPBP* and *PF4*), which were highly regulated and associated with poor survival. Finally, high *DC-STAMP* expression showed a significantly positive correlation with four immune cell [NK CD56 (dim) cells, macrophages, cytotoxic cells, and CD8 (+) T cells] infiltration and high level of immune checkpoint genes (*PDCD1*, *CD274*, *CTLA-4*, and *TIGIT*). Therefore, our results suggest that high expression of *DC-STAMP* predicts adverse outcomes for AML patients.

## Introduction

Acute myeloid leukemia (AML) is a malignant clonal disease originating from hematopoietic stem cells (HSCs) or myeloid progenitors characterized by inhibiting cellular differentiation and proliferation of blast cells (Puram et al., 2016; Assi et al., 2019). Although most patients received traditional chemotherapy and allogeneic hematopoietic stem cell transplantation (allo-HSCT), more than 70% of the patients failed to achieve the desired effects (Chen et al., 2019). Based on some genetic abnormalities, the risk stratification system of AML has been refined and some AML patients tend to have deeper remission and longer survival through molecular targeted therapy (Estey, 2016; Stone et al., 2017; Kayser and Levis, 2018; DiNardo et al., 2020). However, there are limited numbers of reliable biomarkers for indicating the prognosis of AML and guiding therapy selection (Campos et al., 1993; Tzifi et al., 2012; Fröhling et al., 2002; Patel et al., 2012). Therefore, a much more in-depth and comprehensive research of the molecular abnormalities including genetic mutations and validation would aid in designing effective targeted therapies for AML.

Dendritic cell (DC)-specific transmembrane protein (DC-STAMP), also called TM7SF4, is a seven-transmembrane receptor protein, which is predominantly expressed in myeloid DC, macrophages, and osteoclasts (Hartgers et al., 2000; Yagi et al., 2005). However, DC-STAMP mRNA expression is low in hematopoietic stem and progenitor cells (HSCPs) and monocytes (Eleveld-Trancikova et al., 2008). It plays a role in the limitation of myeloid cell differentiation, regulation of the antigen presentation activity of DC, and maintenance of immune tolerance (Eleveld-Trancikova et al., 2008; Sawatani et al., 2008; Eleveld-Trancikova et al., 2010). A recent study demonstrated that the DC-STAMP was considered as an important molecule promoting the development and progression of multiple myeloma (Silvestris et al., 2011), whereas its role in AML is completely unknown.

In this research, we used not only the cox regression analysis but also a nomogram model**,** calibration curves, and a decision curve analysis (DCA) to assess the predictive effect of the DC-STAMP on AML patients based on TCGA database. In addition, we performed three types of enrichment analyses, protein–protein interaction (PPI), and a correlation analysis of immune infiltration or immune checkpoints to detect the pathogenic molecular mechanisms of the *DC-STAMP*. Our findings revealed the prognostic value of the DC-STAMP and may provide novel insights into the gene marker of leukemogenesis.

## Materials and Methods

### Data Source

The transcripts per million (TPM) reads format RNA-seq data of TCGA and GTEx were collected by the toil process from the UCSC XENA browser (https://xenabrowser.net/datapages/) (Vivian et al., 2017; Consortium, 2020; Goldman et al., 2020). The data of 173 cases of AML patients and 70 cases of normal people were extracted from TCGA’s LAML project and GTEx, respectively. The RNA-seq data of the TPM format was performed for an intrasample comparison after log2 transformation. The clinical data of AML patients were downloaded from TCGA (https://tcga-data.nci.nih.gov/), and 153 patients’ data were eligible for inclusion by removing patients without clinical data.

### Differential Gene Expression Analysis

We used the median values of DC-STAMP mRNA expression to divide the AML patients into low and high DC-STAMP expression groups. The differentially expressed genes (DEGs) of the aforementioned two groups were identified by comparing the RNA-seq data of the HTSeq-count format by the DESeq2R package (Love et al., 2014). DEGs were defined as an absolute log2 fold change (|log2 FC|) >1.0 with an adjusted *p* value < 0.05.

### Functional and Pathway Enrichment Analysis

The Gene Ontology (GO) functional gene annotation analysis is a common method used for the enrichment analysis of large-scale genes, including the biological process (BP), cellular component (CC), and molecular function (MF) (Gene Ontology Consortium, 2021). The Kyoto Encyclopedia of Genes and Genomes (KEGG) is a widely used database for information storage of genomes, biological pathways, and diseases and drugs (Kanehisa et al., 2021). We performed the GO and KEGG analyses of DEGs in AML using the R package clusterProfiler (Yu et al., 2012).

### Gene Set Enrichment Analysis

GSEA is a method that evaluates the correlation between gene expression and phenotype from a pre-defined gene set and determines the relative contribution (Subramanian et al., 2005). We downloaded hallmark gene sets (h.all.v7.2. symbols.gmt) from the MsigDB and utilized the R package clusterProfiler to conduct the GSEA (Yu et al., 2012; Szklarczyk et al., 2019). It was considered a statistical significance when the *p* value was less than 0.05.

### Construction of Protein–Protein Interaction Network

Known proteins and predicted protein–protein interaction were investigated by using the STRING website (https://string-db.org/) (Szklarczyk et al., 2019), which contained 9.6 million proteins and 138 million protein–protein interactions from 2,031 species. In this research, we used the STRING database to construct a PPI network of encoding DEGs, then visualized the results and screened hub genes using the Cytoscape software (version 3.7.1) (Shannon et al., 2003). We further performed the ggplot2 package in R to investigate the association of *DC-STAMP* expression with hub genes by the correlation heatmap.

### Correlation Analysis of Immune Infiltration and Immune Checkpoint Genes

We applied the ssGSEA algorithm from the GSVA package (version 1.34.0) to estimate the Pearson correlation coefficient between *DC-STAMP* expression and immune cells and the association of the *DC-STAMP* with the abundance of the 24 types of infiltrated immune cells (Bindea et al., 2013; Hänzelmann et al., 2013). The involved immune cells were activated dendritic cells (aDCs), B cells, CD8 (+) T cells, cytotoxic cells, DCs, eosinophils, immature DCs (iDCs), macrophages, mast cells, neutrophils, NK CD56 (bright) cells, NK CD56 (dim) cells, NK cells, plasmacytoid DCs (pDCs), T cells, T helper cells, T central memory (Tcm) cells, T effector memory (Tem) cells, T follicular helper (Tfh) cells, T gamma delta (Tgd) cells, Th1 cells, Th17 cells, Th2 cells, and regulatory T (Treg) cells. We next performed the ggplot2 package in R to investigate the association of *DC-STAMP* expression with specific immune cells and widely discussed immune checkpoint genes (*PDCD1*, *CD274*, *CTLA-4*, *LAG-3*, *TIGIT*, and *HAVCR2*) by the scatter plot.

### Statistical Methods

**FIGURE 1:**
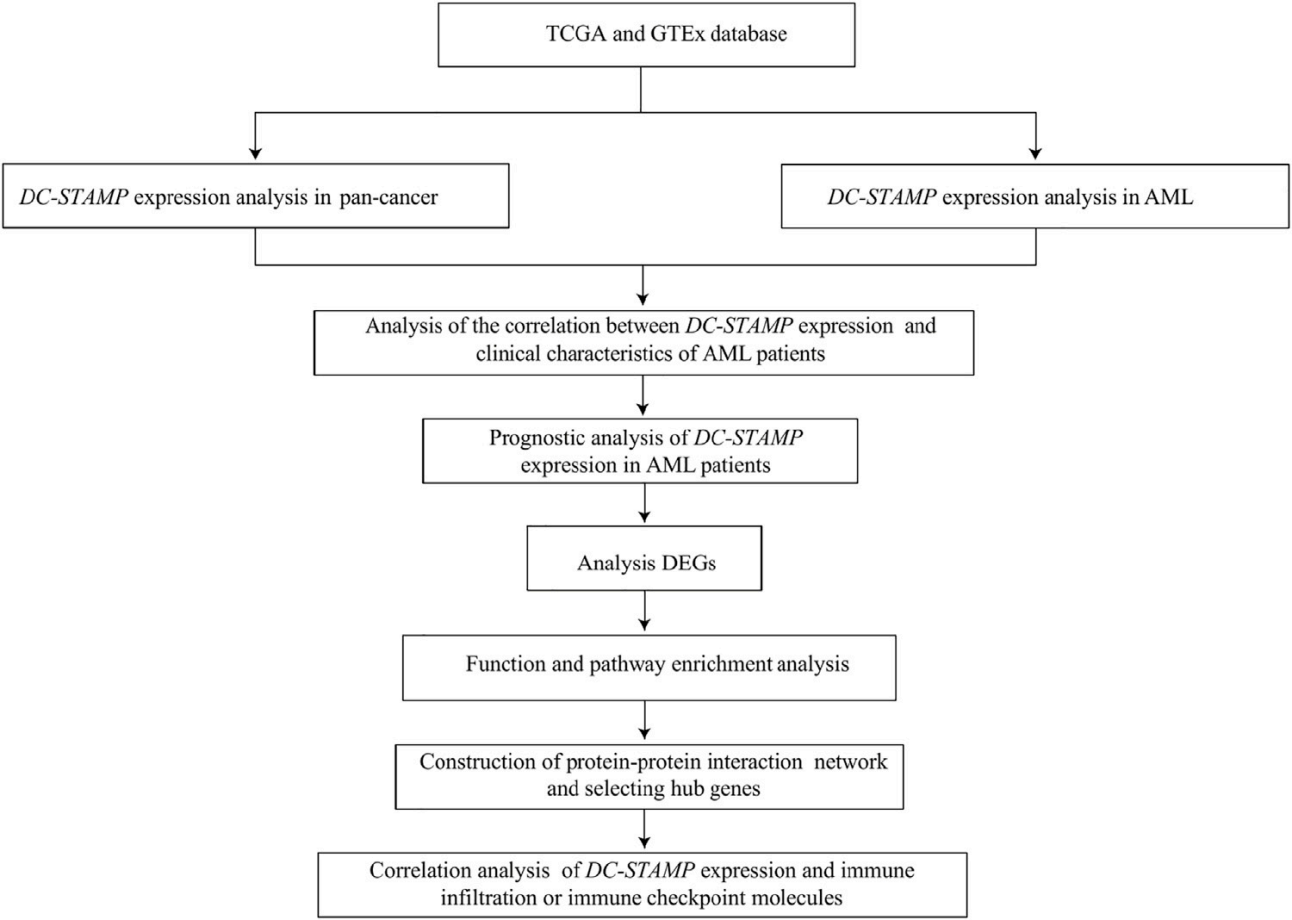
Flowchart of research.

All statistical analyses were completed in R programming (https://www.r-project.org/, version 3.6.3). The effectiveness of the *DC-STAMP* in distinguishing AML from normal samples was assessed by the receiver operating characteristic (ROC) curve analysis using the pROC software. The difference between clinical features and *DC-STAMP* expression was detected by Wilcoxon rank sum tests and Kruskal–Wallis tests. The correlation of clinical features between low and high *DC-STAMP* expression was performed by the Pearson χ^2^ test. Survival curves were constructed using the Kaplan–Meier (KM) plot. The prognostic risk factors were identified by univariate analyses and the multivariate Cox regression analysis, then, independent factors were recruited for building the final nomogram prognostic model. Additionally, we used calibration and DCA to assess the predictive power of the nomogram model. The nomogram plot and calibration curve were established by using the RMS package in R and the DCA curve was constructed by using the survival package and stdca.R. in R. All tests were two-sided, and *p* < 0.05 was considered to be of statistical significance. The research and analysis flowchart is presented in Figure 1.

## Results

### High Expression of *DC-STAMP* is Linked to Unfavorable Clinical Characteristics in Acute Myeloid Leukemia

**FIGURE 2:**
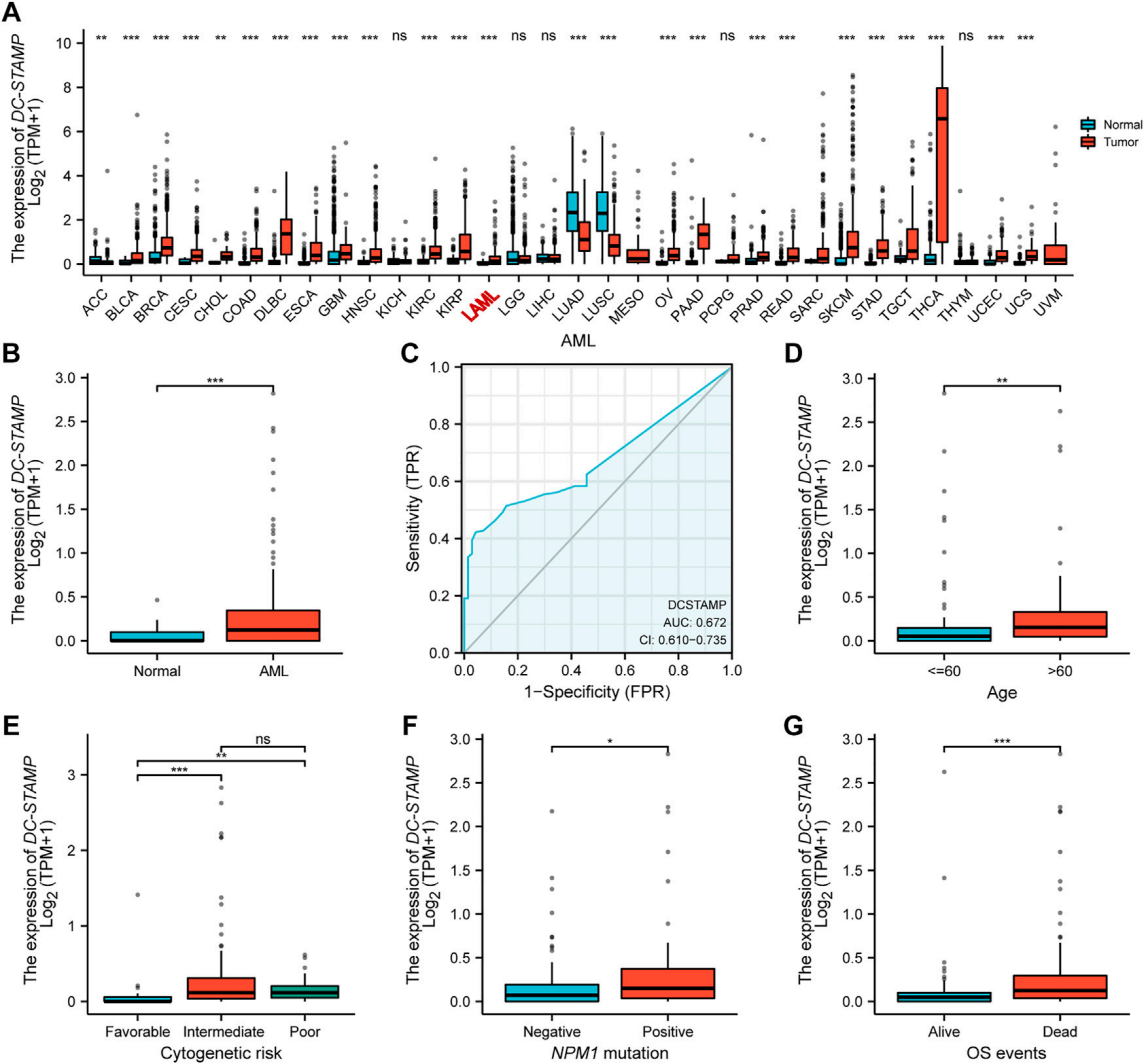
High *DC-STAMP* expression was related with adverse clinical features. **(A)** Level of *DC-STAMP* expression in different tumors from TCGA and GTEx database. **(B)** Expression levels of *DC-STAMP* in AML (*n* = 173) and normal samples (*n* = 70). **(C)** Receiver operating characteristic analysis (ROC) of *DC-STAMP* in AML. Clinical characteristics including **(D)** age, **(E)** cytogenetic risk classification, **(F)** *NPM1* mutation, and **(G)** OS events (*n* = 153). Analysis between two groups of unpaired samples: Wilcoxon rank sum test, analysis among multiple groups of samples: Kruskal–Wallis rank sum test (ns *p* ≥ 0.05, *p < 0.05, **p < 0.01, ***p < 0.001).

We compared the difference in *DC-STAMP* mRNA expression among AML, normal, and other malignancies samples by using the RNA-seq database. Remarkably, *DC-STAMP* expression was upregulated in multiple malignancies (Figure 2A), especially in AML (*p* < 0.05, Figure 2B). Moreover, the power of the expression difference was 0.672 (95% confidence interval, CI = 0.610–0.735, Figure 2C) by the AUC value of the ROC curve analysis. Hence, we are interested in the clinical implications of *DC-STAMP* expression in AML patients. A total of 153 AML patients with clinical information from TCGA were analyzed in the study. As shown in Figures 2D–G, *DC-STAMP* expression was associated with old age (*p* < 0.01, Figure 2D), unfavorable cytogenetic risk (*p* < 0.001, Figure 2E), *NPM1* positive mutation (*p* < 0.05, Figure 2F), and death (*p* < 0.001, Figure 2G), no association with French-American-British (FAB) classifications (Supplementary Figure S1). Furthermore, when patients were grouped by low and high mRNA expression, a strong correlation was found in old age (*p* = 0.004), unfavorable cytogenetic risk category (*p* < 0.001), normal, +8, del (7) karyotype (*p* = 0.008), M2, M5 FAB subtypes (*p* = 0.037), and death (*p* = 0.001, Table 1), but no association with gender, white blood cell count, and *FLT3*, *IDH1*, *RAS*, and *NPM1* mutations. Together, high *DC-STAMP* expression was closely related to poor clinical characteristics.

**TABLE 1:** Clinical characteristics of AML patients with differential *DC-STAMP* expression.

Characteristic	Low expression of *DC-STAMP*	High expression of *DC-STAMP*	*p*
n	75	76	
Age, median (IQR)	51 (39, 62)	61.5 (46.5, 69.25)	0.005^a^
Gender, n (%)			0.084^b^
Female	28 (18.5%)	40 (26.5%)	
Male	47 (31.1%)	36 (23.8%)	
Race, n (%)			1.000^c^
Asian	0 (0%)	1 (0.7%)	
Black or African–American	7 (4.7%)	6 (4%)	
White	67 (45%)	68 (45.6%)	
Age, n (%)			**0.004** ^b^
≤60	53 (35.1%)	35 (23.2%)	
>60	22 (14.6%)	41 (27.2%)	
WBC count (x10^9/L), n (%)			0.255^b^
≤20	34 (22.7%)	43 (28.7%)	
>20	40 (26.7%)	33 (22%)	
Cytogenetic risk, n (%)			**< 0.001** ^b^
Favorable	26 (17.4%)	5 (3.4%)	
Intermediate	34 (22.8%)	48 (32.2%)	
Poor	15 (10.1%)	21 (14.1%)	
FAB classifications, n (%)			**0.037** ^b^
M0	8 (5.3%)	7 (4.7%)	
M1	18 (12%)	17 (11.3%)	
M2	13 (8.7%)	25 (16.7%)	
M3	13 (8.7%)	2 (1.3%)	
M4	16 (10.7%)	13 (8.7%)	
M5	5 (3.3%)	10 (6.7%)	
M6	1 (0.7%)	1 (0.7%)	
M7	0 (0%)	1 (0.7%)	
Cytogenetics, n (%)			**0.008** ^b^
Normal	30 (22.2%)	39 (28.9%)	
+8	2 (1.5%)	6 (4.4%)	
del (5)	1 (0.7%)	0 (0%)	
del (7)	2 (1.5%)	4 (3%)	
inv (16)	5 (3.7%)	3 (2.2%)	
t (15; 17)	10 (7.4%)	1 (0.7%)	
t (8; 21)	7 (5.2%)	0 (0%)	
t (9; 11)	1 (0.7%)	0 (0%)	
Complex	12 (8.9%)	12 (8.9%)	
*FLT3* mutation, n (%)			0.441^b^
Negative	54 (36.7%)	48 (32.7%)	
Positive	20 (13.6%)	25 (17%)	
*IDH1* R132 mutation, n (%)			0.939^b^
Negative	66 (44.3%)	70 (47%)	
Positive	7 (4.7%)	6 (4%)	
*IDH1* R140 mutation, n (%)			0.745^b^
Negative	70 (47%)	67 (45%)	
Positive	5 (3.4%)	7 (4.7%)	
*IDH1* R172 mutation, n (%)			0.245^c^
Negative	75 (50.3%)	72 (48.3%)	
Positive	0 (0%)	2 (1.3%)	
*RAS* mutation, n (%)			1.000^c^
Negative	71 (47.3%)	71 (47.3%)	
Positive	4 (2.7%)	4 (2.7%)	
*NPM1* mutation, n (%)			0.237^b^
Negative	62 (41.3%)	55 (36.7%)	
Positive	13 (8.7%)	20 (13.3%)	
OS events, n (%)			**< 0.001** ^b^
Alive	38 (25.2%)	16 (10.6%)	
Dead	37 (24.5%)	60 (39.7%)	

Bold indicates *p* value less than 0.05

a Derived from the Wilcoxon rank sum test.

b Derived from Pearson’s chi-squared test.

c Derived from Fisher’s exact test.

### High *DC-STAMP* Expression Predicts Worse Prognosis

**FIGURE 3:**
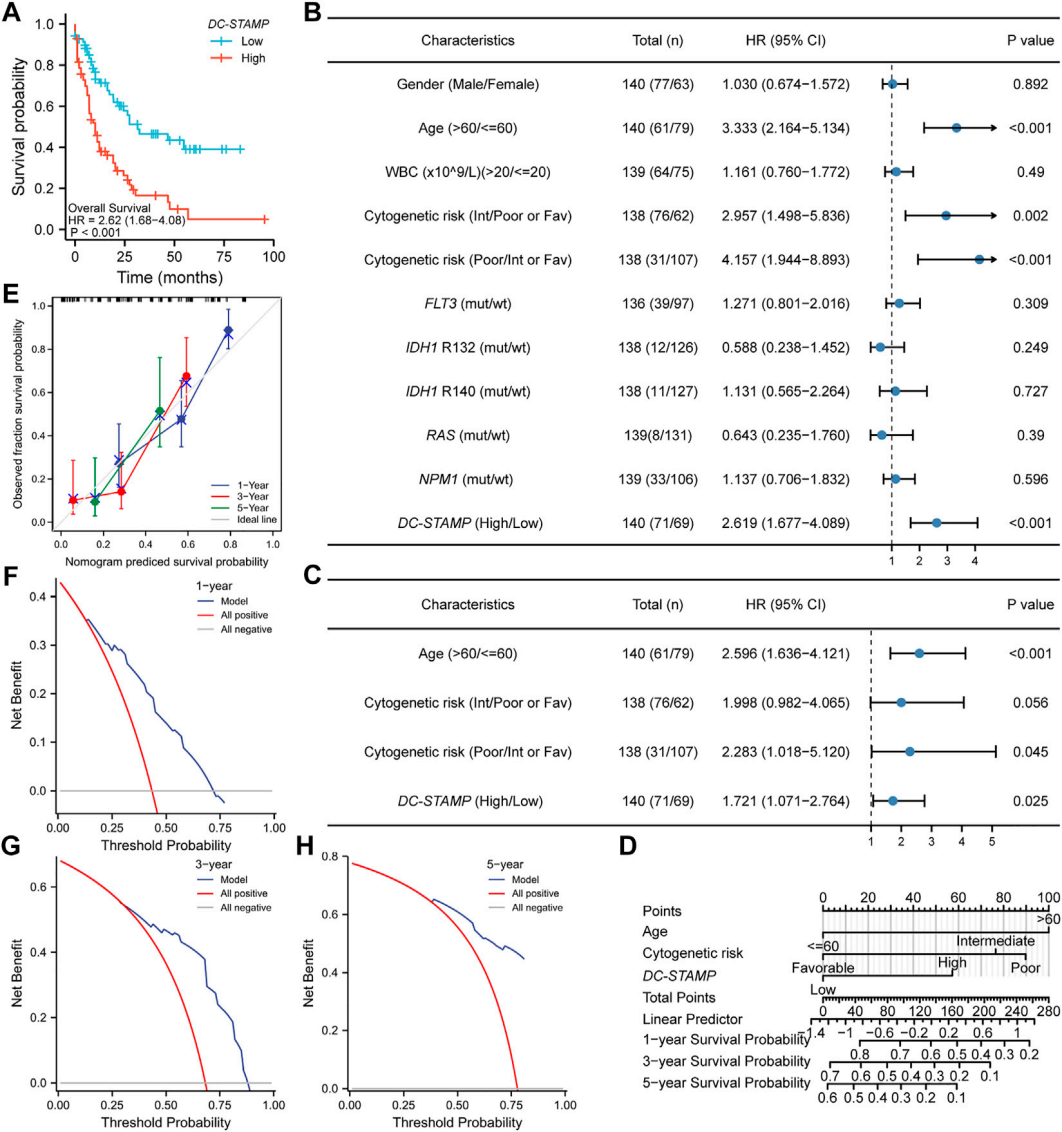
Poor prognostic value of *DC-TAMP* expression in AML. **(A)** KM curve analysis of overall survival (OS) between high and low *DC-STAMP* expression groups. **(B)** Univariate analyses of OS showed by forest plot. **(C)** Multivariate analyses of OS showed by forest plot. **(D)** Nomogram integrates *DC-STAMP* and other prognostic factors in AML. **(E)** Calibration curve of nomogram. The DCA curves of nomogram (F) by 1 year, **(G)** 3 years, and **(H)** 5 years(mut; mutation, wt; wild type, Int; intermediate, Fav; favorable).

We plotted OS curves by the KM method to identify the effect of the *DC-STAMP* on the outcomes in AML patients. Patients with a high expression presented a shorter OS than those with a low expression (*p* < 0.001, Figure 3A). We further use the univariate and multivariate Cox regression analyses to identify the value of the *DC-STAMP* for survival. By the univariate analysis, high *DC-STAMP* expression was associated with shorter OS [hazard radio, (HR): 2.683; 95% confidence interval (CI):1.723–4.178; *p* < 0.001, Supplementary Table S1]. Simultaneously, both age (HR: 3.333; 95% CI: 2.164–5.134; *p* < 0.001) and unfavorable cytogenetic risk (Intermediate: HR: 2.957; 95% CI; 1.498–5.836; *p* = 0.002, Poor: HR: 4.157; 95% CI: 1.944–8.893; *p* < 0.001) were related with poor OS. Then, we included the aforementioned significant univariable factors (*p* < 0.1) in the multivariate analysis and found that age (HR: 2.548; 95% CI: 1.601–4.055; *p* < 0.001), poor cytogenetic risk (HR: 2.293; 95% CI: 1.024–5.135; *p* = 0.044), and high *DC-STAMP* expression (HR: 1.733; 95% CI: 1.079–2.781; *p* = 0.023) were also independent prognostic factors, respectively. In detail, we drew forest plots to present the aforementioned results of the Cox regression analysis (Figures 3B,C, Supplementary Table S1).

Furthermore, to provide a quantitative prediction of the outcomes in AML patients, we constructed a nomogram plot using age, cytogenetic risk, and *DC-STAMP* expression (Figure 3D). The concordance index (C-index) for predicting the OS was 0.734 (95% CI: 0.706–0.762), indicating that the nomogram had a certain predictive accuracy for OS. Additionally, we performed calibration curves and DCA to evaluate the predictive performance of the nomogram model. The calibration curves presented consistency between the predicted OS of the nomogram and the actual proportion of OS at 1-, 3-, and 5-year (Figure 3E). Moreover, the DCA curves also verified the clinical utility of the predictive nomogram (Figures 3F–H). In summary, this nomogram model had an accurate ability to predict the patients’ survival.

### Differentially Expressed Gene Enrichment Analysis Reveals the Dysfunctional Signaling Pathway

**FIGURE 4:**
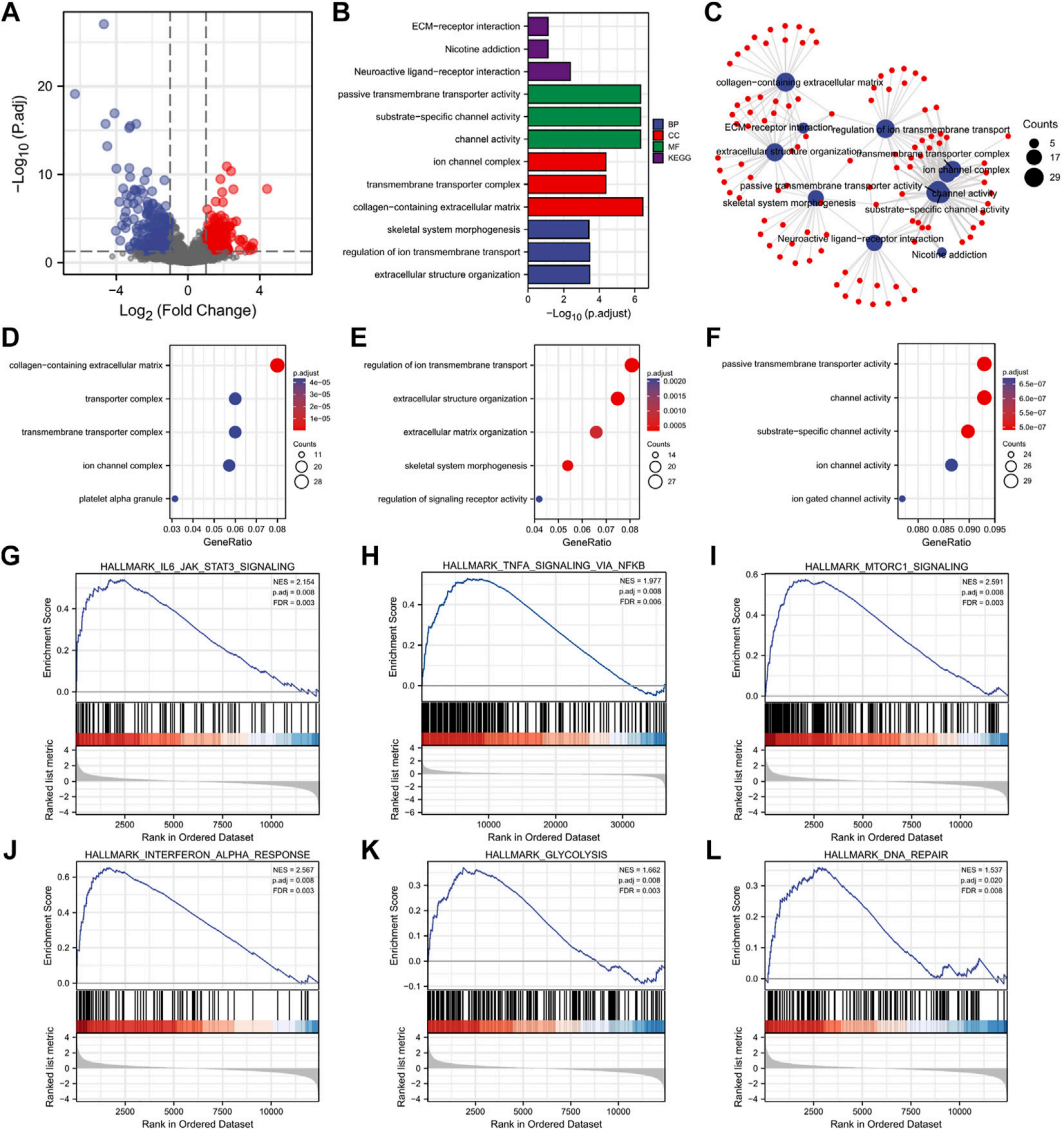
DEG analysis and functional enrichment of high and low *DC-STAMP* expression groups in AML. **(A)** Volcano plot of DEGs. **(B)** GO and KEGG pathway of DEGs. **(C)** Interactive analysis with result of GO and KEGG analyses. **(D–F)** GO enrichment analysis of DEGs. **(G–L)** GSEA of DEGs. **(G)** Enrichment of genes in IL6-JAK-STAT3 signaling pathway. **(H)** Enrichment of genes in inflammatory pathway. **(I)** Enrichment of genes in mTORC1 signaling pathway. **(J)** Enrichment of genes in interferon alpha response pathway. **(K)** Enrichment of genes in glycolysis signaling pathway. **(L)** Enrichment of genes in DNA repair signaling pathway.

We further explored the potential mechanisms in AML patients. Firstly, we identified the DEGs between high and low *DC-STAMP* expression. In total, 610 DEGs were obtained and shown in volcano plots (Figure 4A), including 260 upregulated genes and 350 downregulated genes (|log2FC| >1, adjusted *p* value < 0.05).

Next, we performed the GO and KEGG analyses to investigate the biological function of the aforementioned DEGs and obtained the top 15 GO enrichment items (CC five items, BP five items, and MF five items) and top five KEGG pathways (Figures 4B,D–F and Supplementary Table S2). Briefly, the channel activity, platelet alpha granule, extracellular structure organization, regulation of ion transmembrane transport, neuroactive ligand-receptor interaction, and extracellular matrix (ECM)–receptor interactions were the most enriched sets.

Moreover, to better understand the mutual connection, we performed an interactive analysis derived from the results of GO and KEGG analyses. It showed that the numbers of enrichment genes were channel activity (counts = 29), passive transmembrane transporter activity (counts = 29), collagen-containing extracellular matrix (counts = 28), substrate-specific channel activity (counts = 28), regulation of ion transmembrane transport (counts = 27), extracellular structure organization (counts = 25), transmembrane transporter complex (counts = 21), ion channel complex (counts = 20), neuroactive ligand-receptor interaction (counts = 19), and skeletal system morphogenesis (counts = 18) (Figure 4C).

Finally, the GSEA was used to investigate the enrichment pathway of *DC-STAMP* expression, and a total of 28 significant pathways were enriched (Table 2). Interestingly, some pathways, such as IL6-JAK-STAT3 signaling, mTORC1 signaling, TNF-α signaling *via* NF-κB, INF-γ response, glycolysis, and DNA repair (Figures 4J–L) were reported to correlate with leukemogenesis (Steelman et al., 2008; Park et al., 2010; Binder et al., 2018; Molina et al., 2018; Gabellier et al., 2020; Grants et al., 2020).

**TABLE 2:** Twenty-eight items of GSEA.

Description	Set size	Enrichment score	NES	p. adjust
HALLMARK_EPITHELIAL_MESENCHYMAL_TRANSITION	195	−0.54676	−2.25033	0.008003
HALLMARK_ESTROGEN_RESPONSE_EARLY	200	−0.54257	−2.23437	0.008003
HALLMARK_MYOGENESIS	196	−0.47064	−1.9364	0.008003
HALLMARK_UV_RESPONSE_DN	142	−0.52416	−2.07013	0.008003
HALLMARK_ANGIOGENESIS	36	−0.55865	−1.75389	0.008003
HALLMARK_MYC_TARGETS_V2	55	0.665077	2.430489	0.008003
HALLMARK_ESTROGEN_RESPONSE_LATE	199	−0.39097	−1.60534	0.008003
HALLMARK_IL6_JAK_STAT3_SIGNALING	87	0.541276	2.153534	0.008003
HALLMARK_UNFOLDED_PROTEIN_RESPONSE	110	0.42742	1.766212	0.008003
HALLMARK_INTERFERON_ALPHA_RESPONSE	83	0.654209	2.566687	0.008003
HALLMARK_SPERMATOGENESIS	123	0.48507	2.043111	0.008003
HALLMARK_ALLOGRAFT_REJECTION	199	0.554861	2.511181	0.008003
HALLMARK_MITOTIC_SPINDLE	180	0.502551	2.25991	0.008003
HALLMARK_COMPLEMENT	187	0.365865	1.643381	0.008003
HALLMARK_E2F_TARGETS	187	0.773265	3.473328	0.008003
HALLMARK_G2M_CHECKPOINT	197	0.75184	3.382364	0.008003
HALLMARK_INFLAMMATORY_RESPONSE	194	0.464461	2.091942	0.008003
HALLMARK_GLYCOLYSIS	186	0.370532	1.661545	0.008003
HALLMARK_INTERFERON_GAMMA_RESPONSE	177	0.588205	2.629924	0.008003
HALLMARK_MTORC1_SIGNALING	196	0.576452	2.590705	0.008003
HALLMARK_MYC_TARGETS_V1	193	0.621926	2.792147	0.008003
HALLMARK_TNFA_SIGNALING_VIA_NFKB	198	0.370429	1.666859	0.008003
HALLMARK_UV_RESPONSE_UP	156	0.359304	1.590277	0.014397
HALLMARK_COAGULATION	136	−0.39733	−1.56144	0.017883
HALLMARK_DNA_REPAIR	136	0.358661	1.537167	0.019802
HALLMARK_BILE_ACID_METABOLISM	99	−0.4003	−1.49457	0.034754
HALLMARK_CHOLESTEROL_HOMEOSTASIS	62	0.396308	1.470851	0.036109

### Identification of Hub Genes Connected With *DC-STAMP*

**FIGURE 5:**
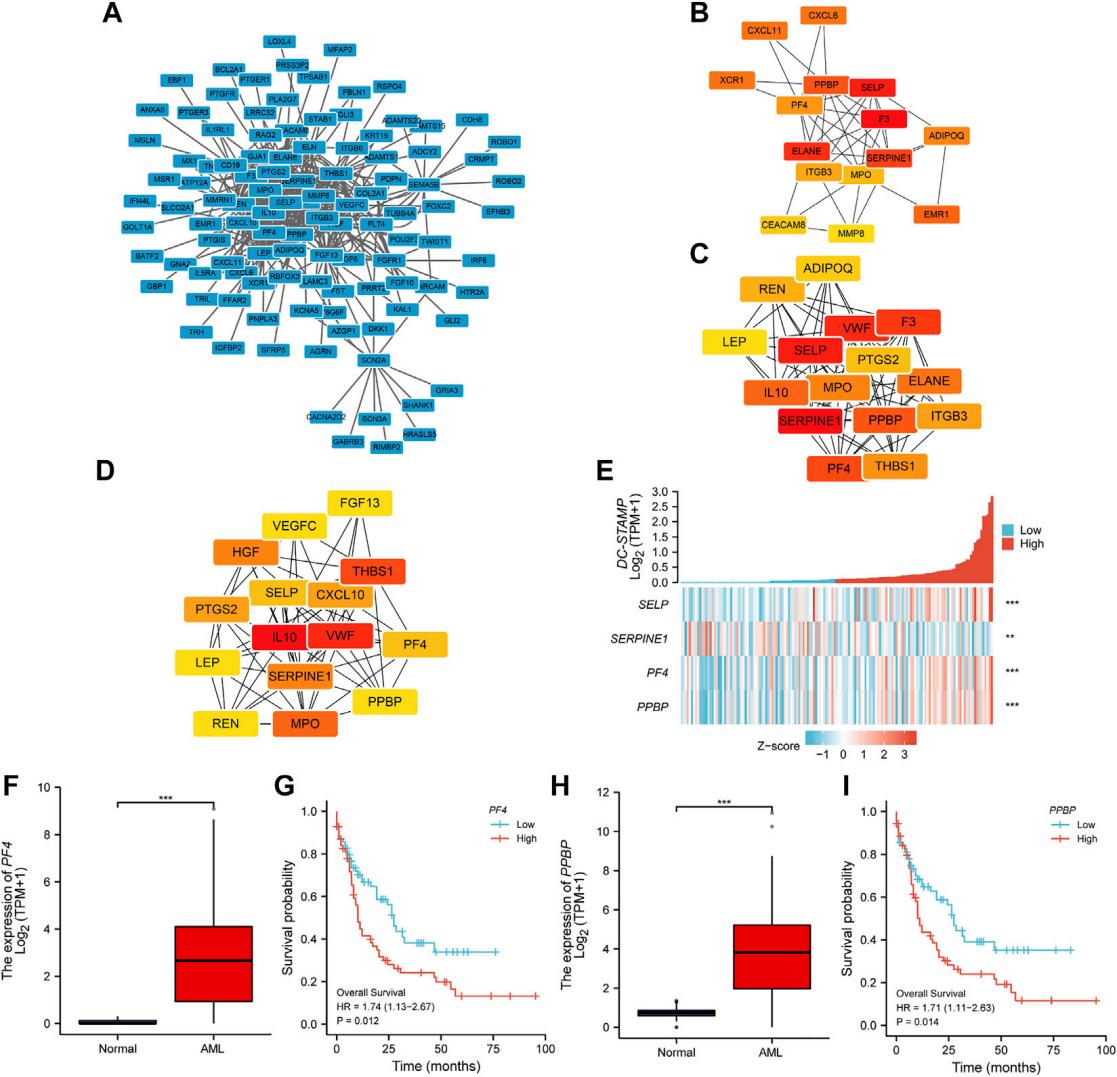
PPI network construction and clinical significance of hub genes. **(A)** The PPI network of 358 encoding DEGs. **(B–D)** Top 15 hub genes were selected respectively by **(B)** MNC, **(C)** DMNC, and **(D)** MCC. **(E)** Association of *DC-STAMP* with four hub genes (*SELP*, *SFRPINE1*, *PF4*, and *PPBP*). **(F)** Expression levels of *PF4* in AML (*n* = 173) and normal subjects (*n* = 70). **(G)** Different OS between high- and low-*PF4* expression levels shown by KM curves. **(H)** Expression levels of *PPBP* in AML (*n* = 173) and normal subjects (*n* = 70). **(I)** Different OS between high- and low-*PPBP* expression levels shown by KM curves. (*p < 0.05, **p < 0.01, ***p < 0.001).

As indicated in Figure 5A, the PPI network of 358 encoding DEGs was constructed to determine the hub genes. The top 15 hub genes were selected by the maximum neighborhood component (MNC), density of maximum neighborhood component (DMNC), and maximal clique centrality (MCC) algorithms, respectively (Figures 5B–D). Therefore, we observed four hub genes (*SELP*, *SERPINE1*, *PF4,* and *PPBP*) shared from the aforementioned three gene lists. We analyzed the association between the *DC-STAMP* and four hub genes. It indicated that the *DC-STAMP* has significant positive correlations with *SELP* (*p* < 0.001, correlation coefficient: 0.364), *PF4* (*p* < 0.001, correlation coefficient: 0.39), and *PPBP* (*p* < 0.001, correlation coefficient: 0.406) (Figure 5E). In contrast, the *DC-STAMP* and *SERPINE1* were negatively correlated (*p* = 0.005, correlation coefficient: 0.228) (Figure 5E). Finally, an analysis of the relationship between the four hub genes and clinical prognosis in AML patients revealed that only *PF4* and *PPBP* were expressed at a high level, which was associated with poor outcomes (Figures 5F–I).

### Correlation Analysis Between *DC-STAMP* and Immune Cell or Immune Checkpoint Molecules

**FIGURE 6:**
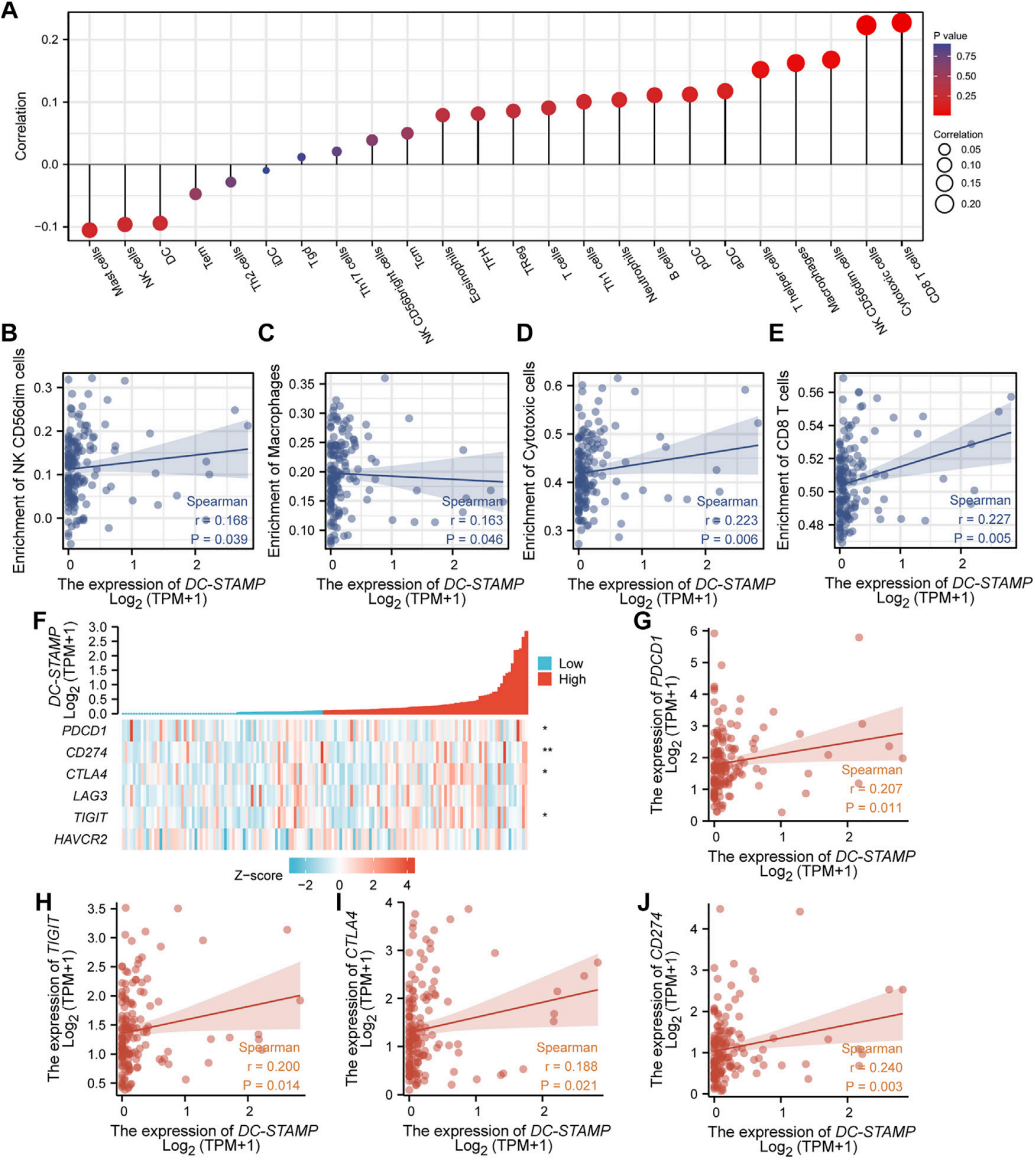
Correlation analysis between the level of *DC-STAMP* gene expression and immune cell infiltration or immune checkpoint molecules. **(A)** Association between *DC-STAMP* expression and 24 kinds of infiltrated immune cells. **(B–E)** Using Spearman’s correlation in quantified analysis of the correlation of *DC-STAMP* expression with infiltrating level of (**B**) NK CD56 (dim) cells, **(C)** macrophages, **(D)** cytotoxic cells and **(E)** and CD8 (+) T cells. **(F)** Association of *DC-STAMP* with five immune checkpoint molecules (*PDCD1*, *CD274*, *CTLA-4*, *LAG-3*, *TIGIT*, and *HAVCR2*). **(G–J)** Using Spearman’s correlation in quantified analysis of the correlation of *DC-STAMP* expression with (**G**) *PDCD1,* **(H)** *TIGIT,* **(I)** *CTLA-4,* and **(J)** *CD274* (r was Spearman’s correlation coefficient) (*p < 0.05, **p < 0.01, ***p < 0.001).

To describe the association of *DC-STAMP* expression with immune infiltration in AML, we systematically evaluated 24 kinds of infiltrated immune cells. The result showed that the level of *DC-STAMP* expression had a significant positive correlation with the infiltrating level of NK CD56 (dim) cells, macrophages, cytotoxic cells, and CD8 (+) T cells (Figure 6A). The details of a quantified analysis by Spearman’s correlation are shown in Figures 6B–E. Furthermore, we analyzed the relationship between *DC-STAMP* expressions and widely discussed immune checkpoint genes (*PDCD1*, *CD274*, *CTLA-4*, *LAG-3*, *TIGIT*, and *HAVCR2*). As shown in Figure 6F, the level of *DC-STAMP* gene expression was significantly and positively correlated with *PDCD1*, *CD274*, *CTLA-4*, and *TIGIT*. The specific correlation analysis is shown in Figures 6G–J.

## Discussion

AML is a hematological neoplastic disease and involves many different molecular genetic abnormalities. The *DC-STAMP* is considered to be a receptor protein, which functions by promoting DC antigen-presentation and osteoclast activation. Only few studies have revealed that overexpression of the *DC-STAMP* would influence the differentiation of myeloid lineage cells (Eleveld-Trancikova et al., 2008). It is also reported that normal HSCPs remain at the low level of the *DC-STAMP* (Eleveld-Trancikova et al., 2008; Eleveld-Trancikova et al., 2010). According to a recent review, a high *DC-STAMP* expression level may have potential pathogenic impacts on myeloid malignancies. However, it is still unknown whether DC-STAMP has an impact on AML.

Based on the aforementioned situation, we first investigated the association between the *DC-STAMP* and clinical features of AML by using TCGA database. As expected, an upregulated *DC-STAMP* expression was observed, and a high level of the *DC-STAMP* gene was correlated with adverse clinical characteristics and poor survival. Thus, it was consistent with the hypothesis that an abnormally high level of *DC-STAMP* expression blocked the differentiation of HSCs in AML patients.

Additionally, we preliminarily explored the pathogenic molecular mechanisms of the *DC-STAMP* by using various bioinformatics analyses. Expectedly, we found DEG enrichment pathways were focused on the molecular transport process and platelet alpha granule by GO and KEGG, while GSEA pathways were involved in mTORC1 signaling, TNF-α signaling *via* NF-κB, and inflammatory and DNA repair pathways. A previous research work reported that the activation of mTORC1 signaling promotes the proliferation and survival of the leukemic clones (Steelman et al., 2008; Park et al., 2010) and cytotoxicity in AML cells from the selective AMPK agonist (GSK621) because of mTORC1 activation which was through the eIF2α/ATF4 signaling pathway (Sujobert et al., 2015). Another study also revealed that the mTORC1 pathway had a correlation with easy relapse and disease progression in AML (Oki et al., 2021). Grants et al. (2020) mentioned that NF-κB, IL6, and TNF were a kind of potential drivers of HSC dysfunction, activating inflammatory signaling in myeloid malignancy. As we know, proinflammatory factors were linked to blast cell growth, and the dysregulation of cytokine signaling contributed to a beneficial AML microenvironment (Binder et al., 2018). Therefore, we think that the effect of the *DC-STAMP* on potential pathogens is probably associated with the aforementioned signaling pathways.

Furthermore, we obtained two hub genes (*PF4* and *PPBP*) with poor OS through different PPI calculation methods and survival analyses. It has been reported that *PF4* and *PPBP* belonged to the CXC chemokine family and played roles in platelet activation, platelet degranulation, immune response to infection, activation of neutrophils and monocytes, and tumorigenesis. (Yan et al., 1994; Martí et al., 2002; Schaffner, 2005; Strieter et al., 2006; Sakurai et al., 2016). These two genes also had been implicated in acute megakaryocytic leukemia, lung adenocarcinoma, gastric cancer, and several autoimmune disorders, including rheumatoid arthritis and Crohn’s disease (Ulivi et al., 2013; Pelleri et al., 2014; Takeyama et al., 2015; Pucci et al., 2016; Xia et al., 2017; Wu et al., 2021). Although the *DC-STAMP, PF4,* and *PPBP* had links with tumor-associated immune response, the mechanisms of the synergistic effects of their interaction remain unclear. A more in-depth detection is needed to explore this complex correlation in AML patients in future.

Finally, when analyzing the relationship between *DC-STAMP* expression and immune cell infiltration, we found that the high *DC-STAMP* group was inclined to harbor more immune cells with cytotoxic effects. As previously demonstrated, the *DC-STAMP* promoted the most efficient CD4 (+) and CD8 (+) T-cell responses *in vitro* (Moulin et al., 2012). Moreover, AML patients had a trend toward increased mature NK cells (NK CD56 (dim) cells) (Tang et al., 2020). We also found *DC-STAMP* expression had a correlation with *PDCD1*, *CD274*, *CTLA-4*, and *TIGIT* which were exhaustion markers of T cells and considered a dysfunction of anti-tumor immunity (Noviello et al., 2019; Wang et al., 2021). A recent study showed that PD1-positive/CD8-positive T cells were higher in relapsed AML patients, compared with newly diagnosed AML patients (Williams et al., 2019). This result may suggest that the *DC-STAMP* was closely related to the immune escape of AML. However, the detailed pathological mechanism of the *DC-STAMP* remains unknown and needs further exploration in the future. The research of *DC-STAMP* expression or the relationship between the *DC-STAMP* and immune checkpoints would be a benefit for the discovery of new immunotherapeutic targets to improve the survival of AML patients.

However, our study still has the following limitations that cannot be ignored. Firstly, we investigate the diagnostic effect of the *DC-STAMP* because of the publicly available TCGA AML database and this observation needs to be subsequently validated in larger independent cohorts. Secondly, although this research comprehensively describes the impact of the *DC-STAMP* level on the survival of AML patients, it lacks the exploration of *DC-STAMP* pathogenic mutations. Lastly, all the interactions between the *DC-STAMP* and AML-associated immune response lack functional validation and detection of the potential molecular mechanisms. Therefore, further laboratory work is required to make up for the aforementioned shortcomings.

## Conclusion

In this research, it was shown that high expression of *DC-STAMP* has an adverse effect on the overall survival of AML patients and is linked to both AML-associated pathway activation and special immune cells or checkpoints, which suggests that high expression of *DC-STAMP* may be a potential independent prognostic factor and an immunotherapeutic target for AML. This finding could help clinicians decide on optimal regimens and explore new targeted therapies for AML patients.

## Data Availability

All data were collected and downloaded from TCGA and GTEx database using the following links: Clinical data of AML was downloaded from TCGA: https://portal.gdc.cancer.gov/projects/TCGA-LAML RNA-seq data of TCGA and GTEx was collected from UCSC XENA browser: https://xenabrowser.net/datapages/.

## Supplementary Material

Supplementary Figure S1 DC*-STAMP* expression has no association with French-American-British (FAB) classifications.

Supplementary Table S1 Univariate and multivariate Cox regression analyses.

Supplementary Table S2 GO enrichment analysis and KEGG pathway analysis.
