# Supplementary material for: High Expression of DC-STAMP Gene Predicts Adverse Outcomes in AML
Source: Front Genet. 2022 Apr 27;13:876689. doi: 10.3389/fgene.2022.876689 (PMC9091727; doi:10.3389/fgene.2022.876689)
Supplement: Supplementary file 1 [file Table1.docx]

**Supplemental Table 1. Univariate and multivariate Cox regression analysis**

| Characteristics | Total(n) | Univariate analysis | |  | Multivariate analysis | |
| --- | --- | --- | --- | --- | --- | --- |
|  |  | Hazard ratio (95% CI) | P value |  | Hazard ratio (95% CI) | P value |
| Gender | 140 |  |  |  |  |  |
| Female | 63 | Reference |  |  |  |  |
| Male | 77 | 1.030 (0.674-1.572) | 0.892 |  |  |  |
| Race | 138 |  |  |  |  |  |
| Asian | 1 | Reference |  |  |  |  |
| Black or African American | 10 | 0.339 (0.038-3.049) | 0.334 |  |  |  |
| White | 127 | 0.468 (0.064-3.393) | 0.452 |  |  |  |
| Age | 140 |  |  |  |  |  |
| <=60 | 79 | Reference |  |  |  |  |
| >60 | 61 | 3.333 (2.164-5.134) | **<0.001** |  | 2.548 (1.601-4.055) | **<0.001** |
| WBC count (x10^9/L) | 139 |  |  |  |  |  |
| <=20 | 75 | Reference |  |  |  |  |
| >20 | 64 | 1.161 (0.760-1.772) | 0.490 |  |  |  |
| Cytogenetic risk | 138 |  |  |  |  |  |
| Favorable | 31 | Reference |  |  |  |  |
| Intermediate | 76 | 2.957 (1.498-5.836) | **0.002** |  | 2.024 (0.998-4.107) | 0.051 |
| Poor | 31 | 4.157 (1.944-8.893) | **<0.001** |  | 2.293 (1.024-5.135) | **0.044** |
| *FLT3* mutation | 136 |  |  |  |  |  |
| Negative | 97 | Reference |  |  |  |  |
| Positive | 39 | 1.271 (0.801-2.016) | 0.309 |  |  |  |
| *RAS* mutation | 139 |  |  |  |  |  |
| Negative | 131 | Reference |  |  |  |  |
| Positive | 8 | 0.643 (0.235-1.760) | 0.390 |  |  |  |
| *DC-STAMP* | 140 |  |  |  |  |  |
| Low | 70 | Reference |  |  |  |  |
| High | 70 | 2.683 (1.723-4.178) | **<0.001** |  | 1.733 (1.079-2.781) | **0.023** |
