# Supplementary figures and images for: High Expression of DC-STAMP Gene Predicts Adverse Outcomes in AML
Source: Front Genet. 2022 Apr 27;13:876689. doi: 10.3389/fgene.2022.876689 (PMC9091727; doi:10.3389/fgene.2022.876689)

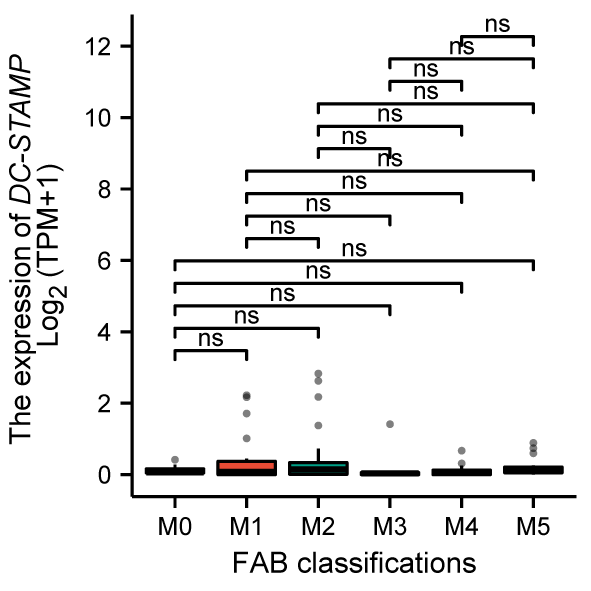

Supplement: Supplementary file 2 [file Image1.tif]
