# Supplementary material for: High Expression of DC-STAMP Gene Predicts Adverse Outcomes in AML
Source: Front Genet. 2022 Apr 27;13:876689. doi: 10.3389/fgene.2022.876689 (PMC9091727; doi:10.3389/fgene.2022.876689)
Supplement: Supplementary file 3 [file Table2.docx]

**Supplemental Table 2. GO enrichment analysis and KEGG pathway analysis**

| ONTOLOGYYonoY | ID | Description | GeneRatio | BgRatio | pvalue | p.adjust | qvalue |
| --- | --- | --- | --- | --- | --- | --- | --- |
| BP | GO:0043062 | extracellular structure organization | 25/334 | 422/18670 | 1.85e-07 | 3.39e-04 | 2.78e-04 |
| BP | GO:0034765 | regulation of ion transmembrane transport | 27/334 | 483/18670 | 1.90e-07 | 3.39e-04 | 2.78e-04 |
| BP | GO:0048705 | skeletal system morphogenesis | 18/334 | 239/18670 | 3.27e-07 | 3.90e-04 | 3.20e-04 |
| BP | GO:0030198 | extracellular matrix organization | 22/334 | 368/18670 | 8.81e-07 | 7.88e-04 | 6.46e-04 |
| BP | GO:0010469 | regulation of signaling receptor activity | 14/334 | 173/18670 | 2.91e-06 | 0.002 | 0.002 |
| CC | GO:0062023 | collagen-containing extracellular matrix | 28/350 | 406/19717 | 1.05e-09 | 3.60e-07 | 2.84e-07 |
| CC | GO:1902495 | transmembrane transporter complex | 21/350 | 324/19717 | 3.80e-07 | 4.32e-05 | 3.41e-05 |
| CC | GO:0034702 | ion channel complex | 20/350 | 301/19717 | 4.89e-07 | 4.32e-05 | 3.41e-05 |
| CC | GO:1990351 | transporter complex | 21/350 | 332/19717 | 5.67e-07 | 4.32e-05 | 3.41e-05 |
| CC | GO:0031091 | platelet alpha granule | 11/350 | 91/19717 | 6.28e-07 | 4.32e-05 | 3.41e-05 |
| MF | GO:0015267 | channel activity | 29/312 | 456/17697 | 2.64e-09 | 4.91e-07 | 4.08e-07 |
| MF | GO:0022838 | substrate-specific channel activity | 28/312 | 428/17697 | 2.70e-09 | 4.91e-07 | 4.08e-07 |
| MF | GO:0022803 | passive transmembrane transporter activity | 29/312 | 457/17697 | 2.77e-09 | 4.91e-07 | 4.08e-07 |
| MF | GO:0005216 | ion channel activity | 27/312 | 416/17697 | 6.23e-09 | 6.72e-07 | 5.58e-07 |
| MF | GO:0022839 | ion gated channel activity | 24/312 | 334/17697 | 6.33e-09 | 6.72e-07 | 5.58e-07 |
| KEGG | hsa04080 | Neuroactive ligand-receptor interaction | 19/153 | 341/8076 | 2.18e-05 | 0.004 | 0.004 |
| KEGG | hsa05033 | Nicotine addiction | 5/153 | 40/8076 | 8.81e-04 | 0.077 | 0.072 |
| KEGG | hsa04512 | ECM-receptor interaction | 7/153 | 88/8076 | 0.001 | 0.077 | 0.072 |
| KEGG | hsa05410 | Hypertrophic cardiomyopathy | 7/153 | 90/8076 | 0.002 | 0.077 | 0.072 |
| KEGG | hsa05414 | Dilated cardiomyopathy | 7/153 | 96/8076 | 0.002 | 0.083 | 0.078 |
